# Zeolite Membranes for Gas Separation: Recent Advances and Challenges

**DOI:** 10.3390/nano16150902

**Published:** 2026-07-23

**Authors:** Qingrun Li, Meijie Jia, Liang Zhu, Changkun Qiu, Chao Fan, Haihan Fan, Tianyi Li, Zhe Yang, Jianye Fu

**Affiliations:** 1 State Key Laboratory of Chemical Safety, SINOPEC Research Institute of Safety Engineering Co., Ltd., National Registration Center for Chemicals, Ministry of Emergency Management, Qingdao 266104, China; liqr.qday@sinopec.com (Q.L.); zhul.qday@sinopec.com (L.Z.); qiuchk.qday@sinopec.com (C.Q.); fanc.qday@sinopec.com (C.F.); 2 State Key Laboratory of Heavy Oil Processing, College of Chemistry and Chemical Engineering, China University of Petroleum, Qingdao 266555, China; z24030060@s.upc.edu.cn (M.J.); 17669642326@163.com (H.F.); litianyi067@gmail.com (T.L.)

**Keywords:** zeolite membranes, fabrication strategies, formation mechanisms, gas separation

## Abstract

Gas separation is a critical industrial process, including natural gas processing, petrochemical production, carbon capture and storage, hydrogen separation, and air separation. Traditional separation methods usually involve high-energy-consuming processes, with inherent limitations in separation efficiency and molecular selectivity. In recent years, zeolite membranes have become an attractive platform for applications such as gas separation, owing to their outstanding selectivity, gas permeability, and energy efficiency. This review systematically summarizes the structural features, representative synthesis strategies, formation pathways, and typical gas separation performances of zeolite membranes. Moreover, existing bottlenecks related to membrane assembly techniques are summarized, and forward-looking perspectives on their further development are put forward. We present several strategic insights, which can provide theoretical guidance for the research and development of zeolite membranes. By providing a comprehensive and timely review, this article aims to promote the application of zeolite membranes in gas separation.

## 1. Introduction

Membrane technology has garnered substantial attention over recent decades due to its low energy consumption, mild operational conditions, and superior separation efficiency for mixtures [1,2,3,4]. Until now, it remains one of the most critical process intensification techniques.

Based on the composition of materials, membranes are generally categorized into polymer membranes, zeolite membranes, mixed matrix membranes, MOFs, etc. Though the polymer membrane is widely used in different separation processes due to its low preparation cost and high reproducibility, the balance between fabrication pollution and performance (e.g., permeance and selectivity) is considered the main challenge in the membrane fabrication field. Inorganic membranes are generally regarded as a promising strategy to overcome the above-mentioned problems, as they have relatively stable and hydrophilic chemical properties, high heat and mechanical stability, and high permeability and selectivity. As a main category of the inorganic membranes, zeolite membranes have attracted great research attention. As is known, zeolites are inorganic crystalline materials with uniform microporous pores and channels, generally comprising silicon–oxygen tetrahedra (SiO_4_), aluminum–oxygen tetrahedra, and other heteroatoms. This architecture results in outstanding molecular sieving performance. Zeolite membranes can efficiently separate liquid and gas mixtures, particularly those with very similar molecular sizes and shapes, due to the molecular sieving capacity of zeolites and the corresponding high adsorption of specific gas molecules.

**Figure 1:**
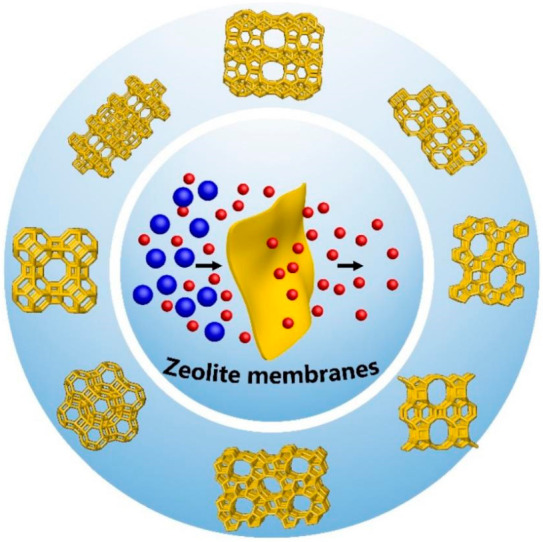
Schematic illustration of zeolite membranes. This review briefly discussed the fabrication of zeolites membranes, as well as strategies to minimize the defects and representative applications of zeolite membranes.

Zeolite membranes have achieved rapid development over the decades since the earliest relevant research was reported. Various types of zeolites have been successfully used for the fabrication of zeolite membranes (Figure 1). Many synthetic strategies have been developed for the fabrication of zeolite membranes, including the self-limiting growth method, multi-parameter-controlled fabrication, gel nuclei-less route, gel-modulated strategy, dry rolling method, molecular layer deposition, etc. These methods are capable of modulating the thickness of the membranes to realize a desired gas permeance and target gas selectivity. Moreover, the separation mechanisms of zeolite microporous structures and their separation performance have been proposed. The inorganic microporous zeolite membranes have exhibited great potential in various important applications, e.g., the reduction in greenhouse gas emission in flue gas, H_2_ separation, CO_2_ recycling from natural gas, and clean water production [5,6]. Though problems still exist, such as synthetic reproducibility, defects on the membrane, manufacturing costs, large-scale fabrication, etc., and zeolite membranes have only been successfully used in a few industrial applications, this has not deterred researchers from pursuing the state-of-the-art assembling strategy to fabricate advanced zeolite membranes and eventually realize their application. Recently, with the growing research attention focused on the use of hydrogen energy and carbon utilization, the application of inorganic zeolite membranes in the field of gas separation has broad prospects and thus become a research hotspot.

Over the past few decades, several reviews have been published regarding the fabrication and separation performance of nano-sized zeolites, focusing on their potentially broad range of diverse applications [3,7]. To date, zeolite materials have been proven effective as adsorbents and separation membranes. Results have revealed that their separation performance primarily depends on zeolite’s pore characteristics. As zeolite materials gain mature applications in gas separation, it is essential to summarize the similarities and differences among various zeolite membranes and clarify the design standards for membrane separation materials. Although significant progress has been made in this field, there is a lack of systematic analysis of the membrane crystallization formation mechanism, inter-crystalline defect regulation, and challenges in large-scale assembly and industrialization implementation. Compared with previous reviews, this summary not only systematically integrates various manufacturing strategies for zeolites but also focuses on discussing their formation mechanisms and the strategies for reducing membrane defects, as well as establishing the correlation between topological structure, chemical composition, and gas separation performance (e.g., H_2_/CO_2_, H_2_/CH_4_, and CO_2_/CH_4_). In addition, this paper prospects emerging research directions, including AI-assisted membrane design and machine-learning-based optimization of synthesis procedures, as well as large-scale fabrication. Discussions on industrial implementations are also presented, offering new insights into fundamental research and future industrial applications of high-performance zeolite separation membranes.

## 2. General Fabrication of Zeolite Membranes

Most zeolites comprise crystal aluminum silicate, and have molecular-level structures with good adsorption performance and high thermal stability and chemical stability. The nanostructure of zeolites endows them with properties to separate gas and liquid mixtures as membranes based on their molecular screening effect and adsorption selectivity [8]. Usually, these zeolite membranes are prepared on porous substrates, as the independent zeolite layer is brittle and without mechanical strength. It has been demonstrated that several methods are currently capable of preparing these supported zeolite-type membranes, mainly growing through one-step or secondary growth [9]. In the one-step hydrothermal system, the porous substrate is immersed in precursor gel for high-temperature crystallization. After the system reaches supersaturation, heterogeneous nucleation on the substrate surface and homogeneous nucleation in the bulk solution proceed simultaneously without stage separation. Crystal nucleation persists throughout the reaction, and newly formed nuclei compete with early nanoparticles for silicon–aluminum precursors to support crystal growth. This synchronous nucleation and growth process is highly sensitive to subtle variations in reaction conditions, which changes the balance between two nucleation pathways and generates inconsistent membrane morphology and defect density across batches [10]. Therefore, the secondary growth method is the most commonly used, as it guarantees better control over zeolite growth and the membrane structure [11]. The secondary growth method encompasses various steps, including synthesizing the seeding cores of zeolite, depositing the seeds onto the substrate surface, and stabilizing them with a growth layer through chemical bonds. For the coating of zeolite seeds on the substrate surface, the interaction between the carrier and the seeding core is determined by the pH value and the related zeta potential of two different materials [12]. Generally, the surface of the substrate is positively charged to improve the static interaction with the zeolite nucleus [13]. Moreover, it is difficult to obtain a uniform layer though only one deposition process. In order to reach this stage, several repeated depositions are required. After the seed deposition method, the secondary growth process influences the thickness and permeability of the zeolite membrane—in a gel-free method, the zeolite membrane becomes almost flawless. This method is cheap and fast, and it is extremely suitable for supporting flat surfaces. By using a microwave oven instead of traditional heating methods, the crystal size is extremely narrowly distributed with reduced defects [14,15]. In 2026, Wang et al. efficiently fabricated intact compact twin-free MFI zeolite membranes via microwave-aided secondary growth over pre-deposited crystalline seed layers [16]. However, the commercial application of microwave heating is limited by the high costs of complex microwave systems [17,18,19,20]. The fabrication of zeolite with different zeolite topologies in various separation processes has remained an area of research for many years. Many research teams have prepared membranes to improve the molecular screening effect with a fixed direction of crystals. For example, a high-oriented MFI zeolite film was prepared by adopting a new way that utilizes a structure-directing agent [21]. And in 2026, Liang et al. developed a fabrication strategy for robust b-oriented pure silica MFI zeolite (Si-MFI) membranes. By tuning the hydrothermal synthesis parameters to suppress homogeneous random nucleation, the selective adsorption of TPA^+^ cations on the b-axis (010) crystal plane was leveraged to facilitate the growth of well-ordered straight-channel membranes with reduced particle boundary defects [22]. These membranes expressed interesting performance in separating chemical mixtures.

However, the large-scale production of directional membranes is still key to developing simple, environmentally friendly, and cost-effective approaches. Another way to reduce defects of zeolite membranes is to use a very thin zeolite layer in the seeding stage of core crystals. This method is time-consuming though. The produced nanosheet zeolite membranes show fascinating performance in terms of their ability to penetrate and separate, but it is difficult to achieve feasible and scalable production methods. In addition, many different synthesis techniques are employed to reduce existing defects in zeolite membranes. For example, chemical gas deposition (CVD) can prevent relatively small defects, while the efficiency is low for larger flaws. In the past few decades, people have conducted a lot of work to develop non-defective zeolite membranes. There is still a long way to go to before such membranes can reach large-scale production.

## 3. Design and Synthesis of Zeolite Membranes

Zeolite membranes with thin zeolite layers have relatively high permeance. There are several successful cases of synthesized ultra-thin zeolite membranes (<1 μm), including silicalite-1, CHA, and SSZ-13 membranes, among others [23,24,25,26,27,28,29]. Zeolite membranes with a reduced thickness of ~100–500 nm generally exhibit advanced separation performance for various mixtures. Notably, silicon CHA films with a thickness of ~500 nm have been prepared by using nano-sized CHA seeds (<100 nm) and a high template concentration. Until now, many fabrication methods were developed for the assembly of zeolite membranes. As shown in Table 1, these methods include the self-limiting growth method, multi-parameter-controlled fabrication, gel nuclei-less route, gel-modulated strategy, dry rolling method, molecular layer deposition, etc. Representative examples are presented in this section in order to illustrate the various fabrication techniques for zeolite-based membranes.

Moreover, as an important part of zeolite membrane fabrication, i.e., reducing surface defects, several strategies have been proposed to eliminate the surface defects and modulating the thickness of the membranes to realize a desired gas permeance and target gas selectivity. All these strategies are presented below.

### 3.1. Self-Limiting Growth Method

**Figure 2:**
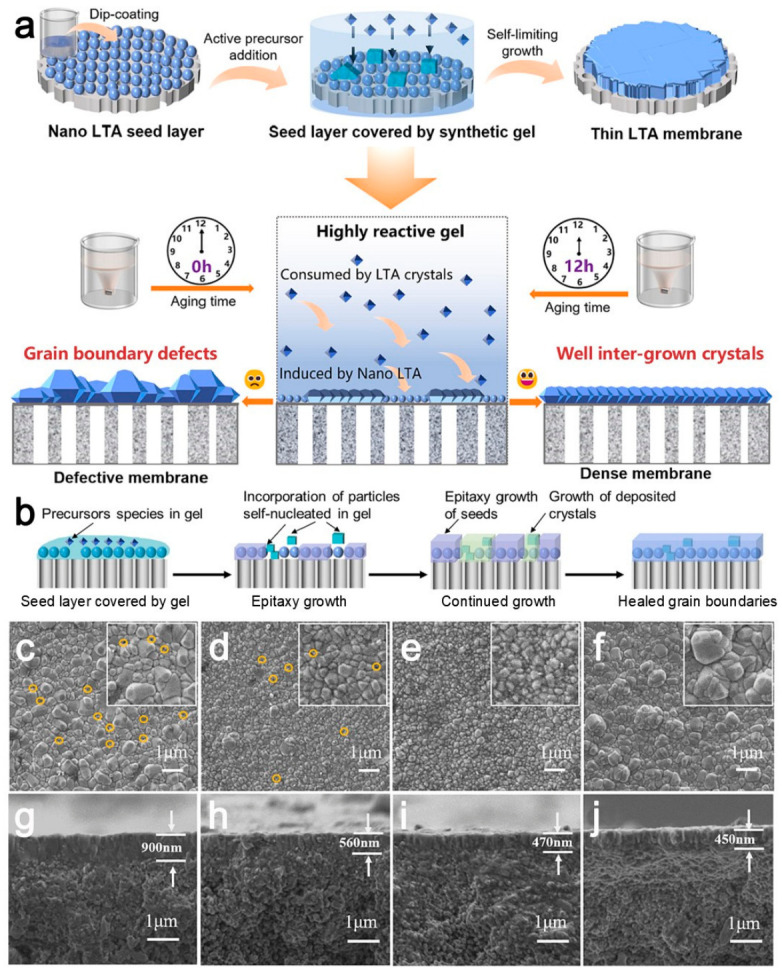
Illustration of the preparation of LTA membranes based on the self-limiting growth approach (**a**) and its corresponding formation mechanisms (**b**). Top and cross-sectional SEM images of LTA membranes with various gel aging times and the corresponding controlled thickness of zeolite layers: 0 h (**c**,**g**), 6 h (**d**,**h**), 12 h (**e**,**i**), and 24 h (**f**,**j**). The yellow circles in c and d indicate the presence of intergranular defects. Reproduced with permission from reference [30]. Copyright 2024, Elsevier.

LTA-type zeolite, featuring a small pore size of 0.4 nm, is widely recognized as one of the most promising materials for constructing zeolite membranes with high H_2_ separation efficiency [36]. However, due to the low silicon–aluminum ratio, the density of LTA zeolite membranes is subject to inevitable interface defects caused by the high electronegativity of zeolite [37]. Generally, due to the existence of crystal defects, the gas diffuses through the LTA film via Knudsen diffusion [38,39]. To mitigate the adverse effects of these crystalline defects, conventional approaches require the fabrication of relatively thick LTA films, which unfortunately results in reduced gas permeability [40,41,42]. Researchers have attempted to reduce the thickness of LTA films to overcome the penetration issue. Zou and others converted the continuous and dense LTA zeolite film through a FAU zeolite film [43]. However, this strategy often yields mixed-phase zeolite films, and the fabrication of ultra-thin, dense single-phase LTA membranes remains a significant challenge for achieving optimal separation performance. Moreover, the high electronegativity of LTA zeolite poses additional hurdles to the preparation of dense, submicron-thick LTA-type membranes. In 2024, Guo et al. [30] prepared a thin and dense LTA film through a self-limiting growth method. This method involved a two-stage growth process of nano-LTA seeds (Figure 2). A uniform nano-sized seed layer helps the continuous growth of the membrane. They used the synergies of high-active gel and nano-sized seeds to control the self-limited growth of the zeolite membrane layer to prepare a film. By optimizing the aging time of gels, they further improved the film density of the membrane through the effective symbiotic growth of zeolite crystals. This represents the first successful synthesis of a single-phase LTA film with a thickness below 1 μm. The key factors governing this synthetic strategy are detailed as follows: High-active gel promotes the propagation of nano-sized seeds, limiting the crystal size during the fabrication process, thereby limiting the thickness of the LTA film in turn. The optimization of the aging process accelerates the consumption of nutrients in the gel, and promotes the growth of crystals. Therefore, finely tuning all these parameters allows for regulating the crystal shape and thickness of the LTA membrane, so as to obtain a high-selective H_2_/CO_2_ and H_2_/CH_4_ separation with a high-permeance membrane.

### 3.2. Multi-Parameter-Controlled Fabrication

**Figure 3:**
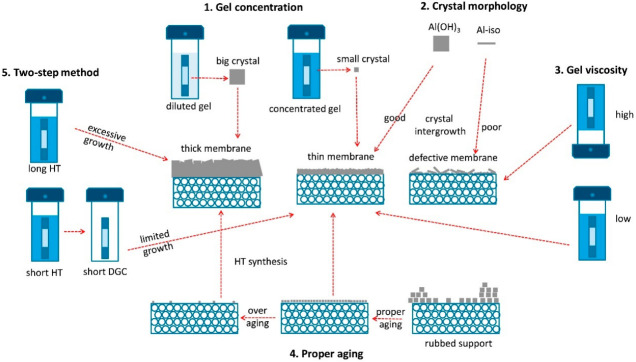
Schematic illustration of synthetic strategies for the fabrication of thin layer SAPO-34 membranes. Reproduced with permission from reference [25]. Copyright 2020, Elsevier.

Compared with the above-mentioned zeolite membranes, the synthesis of ultra-thin SAPO-34 zeolite membranes has been barely explored. To achieve this, in 2020, Zhang et al. prepared uniform SAPO-34 membranes under conditions of high TEAOH/Al_2_O_3_ ratios and low H_2_O/Al_2_O_3_ ratios [25]. A high concentration of the precursor in the synthesized gel promotes the nucleation of zeolite cores, thereby facilitating the generation of small SAPO-34 crystals and enabling a reduction in the membrane layer thickness from 4.3 μm to 1.2 μm (Figure 3). Moreover, after careful optimization through the aging process, the membrane thickness can be reduced to approximately 900 nm. Finally, the multiple parameters controlled fabrication route is applied to reduce the thickness of the membrane by suppressing the uncontrolled growth of the zeolite membrane. As illustrated in Figure 3, multiple fabrication parameters are systematically optimized to minimize the defect density in the SAPO-34 membrane. Firstly, a concentrated zeolite synthetic gel is used with a low H_2_O/Al_2_O_3_ ratio and high TEAOH/Al_2_O_3_ ratio. The concentrated gel enhanced the nucleation process, leading to smaller crystals and a correspondingly thinner membrane [44,45,46,47]. The second parameter is the use of Al(OH)_3_, as it is better than other Al sources; Al(OH)_3_ is conducive to forming cubic-type SAPO-34 crystals in thick gels. Therefore, Al(OH)_3_ promotes the formation of thin plate-shaped SAPO-34 crystals and low-defect zeolite membranes. The third step is to optimize the mixing order. When the viscosity of a membrane gel is decreased, the membrane quality and the reproducibility are greatly improved. Fourthly, appropriate aging of the seeds in the membrane gel is critical to forming a thin and uniform SAPO-34 seed coverage, which is beneficial to forming thin and non-defective membranes. Finally, a two-step process involving hydrothermal treatment and dry gel conversion, which is applied to restrict excessive membrane growth during dry gel transformation. Together, these methods enable the control of submicron SAPO-34 membranes with low defect densities.

### 3.3. “Gel Nuclei-Less” Route

**Figure 4:**
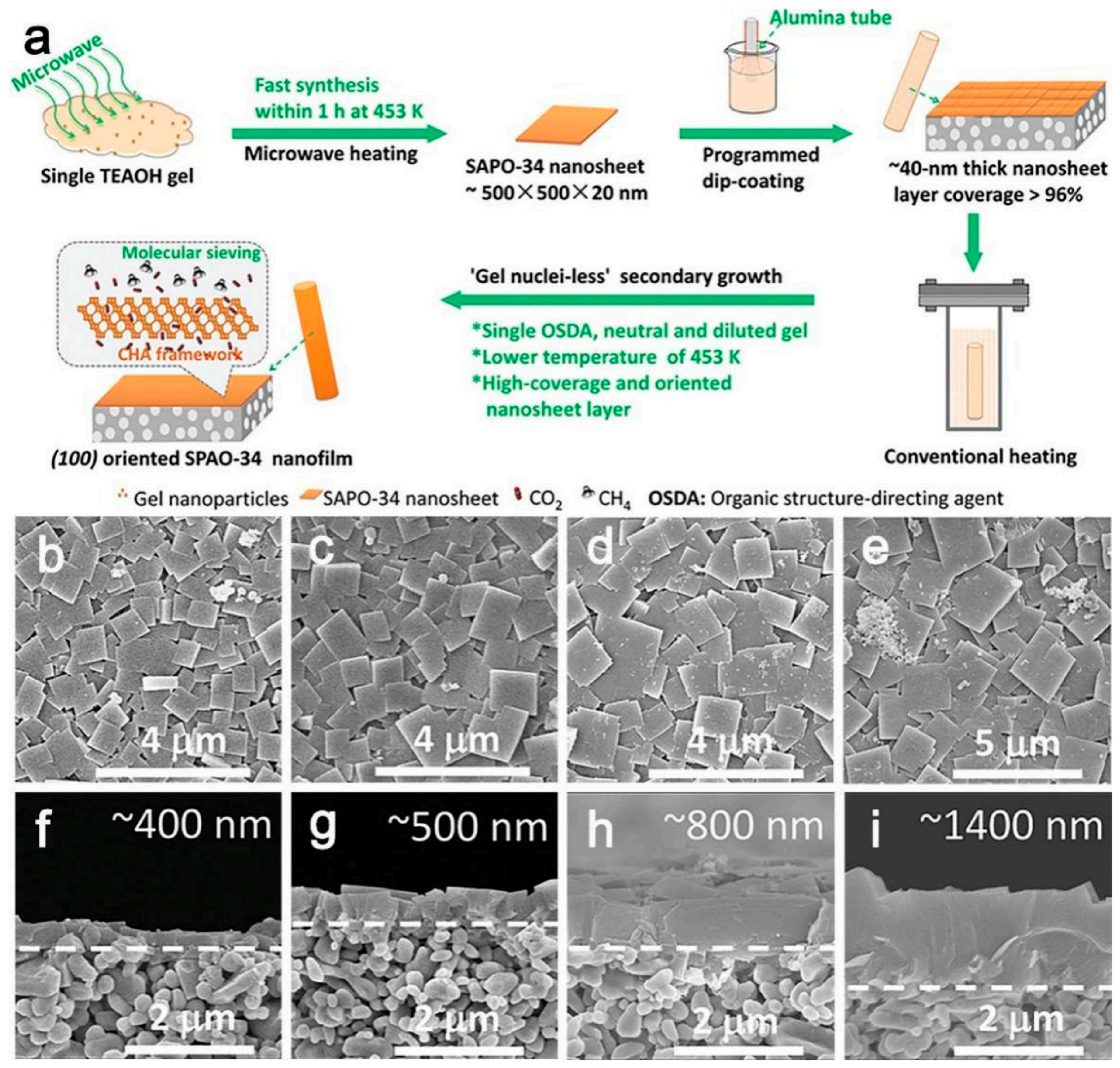
Schematic illustration of zeolite membranes based on the “gel nuclei-less” synthetic strategy (**a**); top and cross-sectional SEM images of the oriented SAPO-34 membranes with various synthesis times: 3 h (**b**,**f**), 6 h (**c**,**g**), 12 h (**d**,**h**), and 18 h (**e**,**i**). The * denote key characteristics of the “gel nuclei-less” secondary growth strategy. Reproduced with permission from reference [31]. Copyright 2020, Wiley-VCH.

Most of the reported SAPO-34 membranes are mostly randomly oriented, with disordered and deformed nano-scale pore structures [48,49,50]. When the zeolite membrane is used in gas separation fields, if ordered, vertical and short nano-channels can be created, and the permeability of specific gases (e.g., CO_2_) are expected to be improved to a higher level. The control of crystal orientation and thickness in continuous crystalline membranes remains challenging due to the inherent difficulty in suppressing spontaneous nucleation. In 2020, Zhou et al. reported a straightforward strategy to control the thickness and orientation of zeolite membranes through the suppression of zeolite nucleation during gel synthesis [31]. As shown in Figure 4, the authors prepared a priority-oriented SAPO-34 nanomaterial derived from SAPO-34 nanocrystals. They rationally designed a simple route to prepare high-quality oriented SAPO-34 nanocrystals, named as the “gel nuclei-less” method (Figure 4a). By optimizing the synthetic parameters to inhibit the formation of the gel, the priority orientation of the nano-sized seed layer is maintained after secondary growth, forming a continuous nanofilm. The ordered and straight nano-channels are then constructed as large-area zeolite crystals without additional defects. This represents a new milestone in the control of zeolite membrane regulation. This “gel nuclei-less” synthetic approach is also applicable to the fabrication of other orientated and ordered nano-channels with thin crystal layer membranes.

### 3.4. Gel-Modulated Strategy

**Figure 5:**
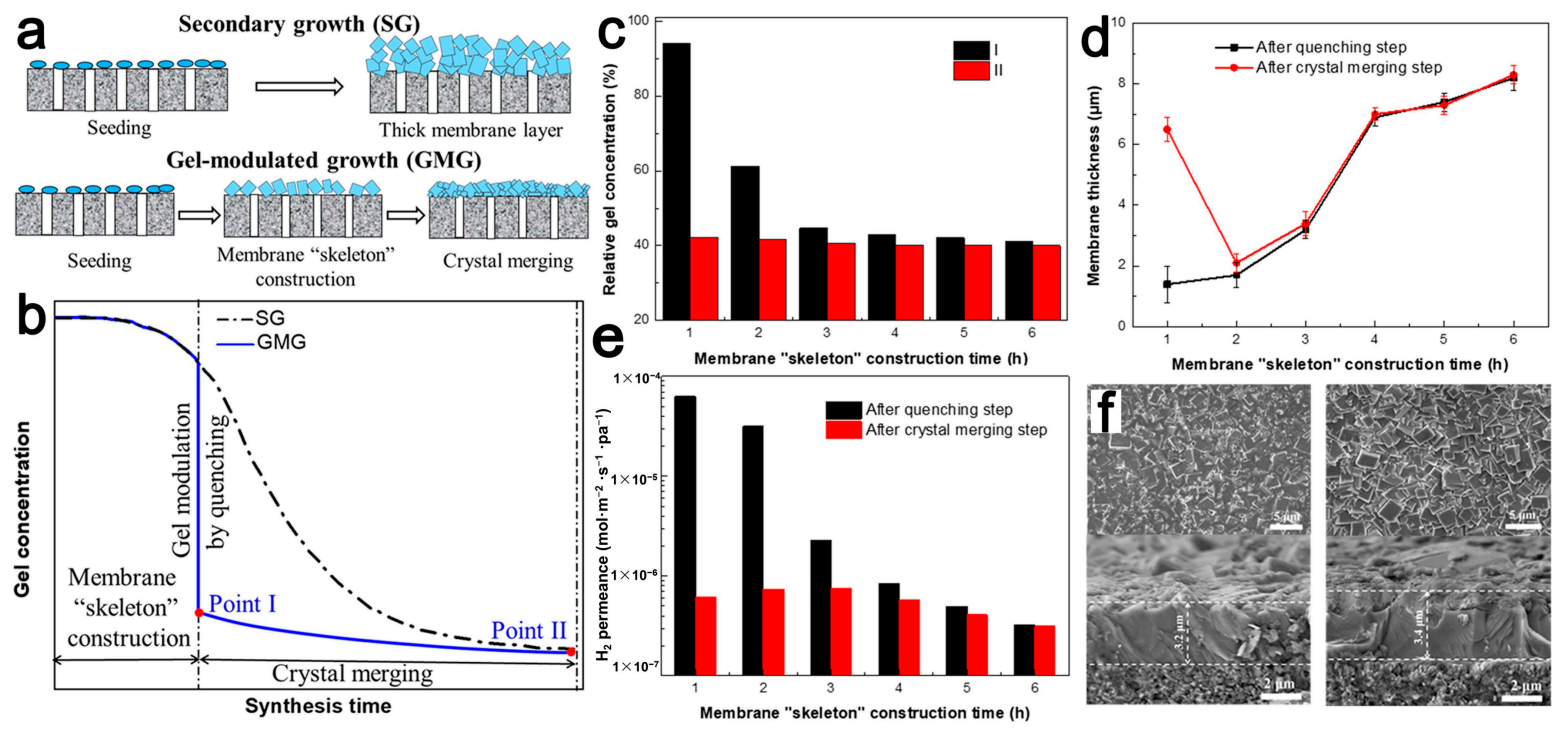
Schematic illustration of the seeded growth process and gel-modulated growth process for the fabrication of SAPO-34 zeolite membranes (**a**); gel concentration change in the seeded growth process along with the synthesis time (**b**); relative gel concentration change (**c**); membrane thickness change along with the construction time (**d**); H_2_ permeance variation along with the construction time (**e**); SEM images of SAPO-34 membranes prepared under different strategies (**f**). Reproduced with permission from reference [32]. Copyright 2020, American Chemical Society.

Zeolite membranes are usually fabricated through the traditional synthetic method, seed growth method, and in situ growth method [51,52,53,54]. In the conventional synthesis method, a synthetic gel with a uniform nutrient concentration is used to generate nuclei and zeolite crystals. However, this is an unavoidable formation due to the volume of research into the deficiency of crystals formed in the high concentrations of synthetic gel. Due to the large size of the nuclei formed, the crystal growth rate is rapid, particularly during the initial stages of synthesis. As a result, thicker membrane layers are usually formed. To form a thin zeolite membrane, the seed growth method has become the most widely used one. The synthetic gel concentration continues to change with the synthesis process time. However, an inadequate seeding process often results in zeolite films with low selectivity, particularly when hollow fiber supports are used. Furthermore, the growth of high-purity zeolite crystalline phases demands a sufficiently high starting gel concentration, while dynamic shifts in gel concentration throughout the synthesis process tend to induce overgrowth during crystal formation [52]. An increase in the seed layer and membrane thickness may also have an impact on the membrane’s performance. To control the growth and crystallization process of zeolite seeds, a gel-modulated strategy was proposed by Yu et al. in 2020 [32]. The new gel-modulated growth strategy is able to prepare high-selective membranes without compromising their permeance property. Specifically, in this strategy, thin high-selective zeolite films were prepared by building a membrane “skeleton” on brackets. The synthetic gel is diluted through quenching, and then the diluted film “skeleton” crystals are merged into the gel. As a result, crystal deficiencies and pinholes can be eliminated during the process of crystal merging by using different crystals. These steps lead to a significant increase in selectivity while membrane transparency is not greatly sacrificed. Figure 5 presents a diagram illustrating the seed growth process alongside the gel-modulated growth process. At the outset, a thin and sparsely dense crystalline layer rapidly develops within the highly concentrated gel. Then, the gel concentration is abruptly decreased through the quenching process. As the quenching temperature suddenly drops, a lack of nutrition for crystal growth occurs in the upper liquid layer. Crystals then grow along the membrane. Finally, further growth/extension of the gap/defect in the existing zeolite crystal structure depends on the availability of nutrients in the limited upper liquid layer of synthetic gel. Therefore, the defects of the membrane “skeleton” and the crystal boundary will be fixed to form a thin high-selective zeolite membrane, as illustrated in Figure 5f.

### 3.5. Dry Rolling Method

**Figure 6:**
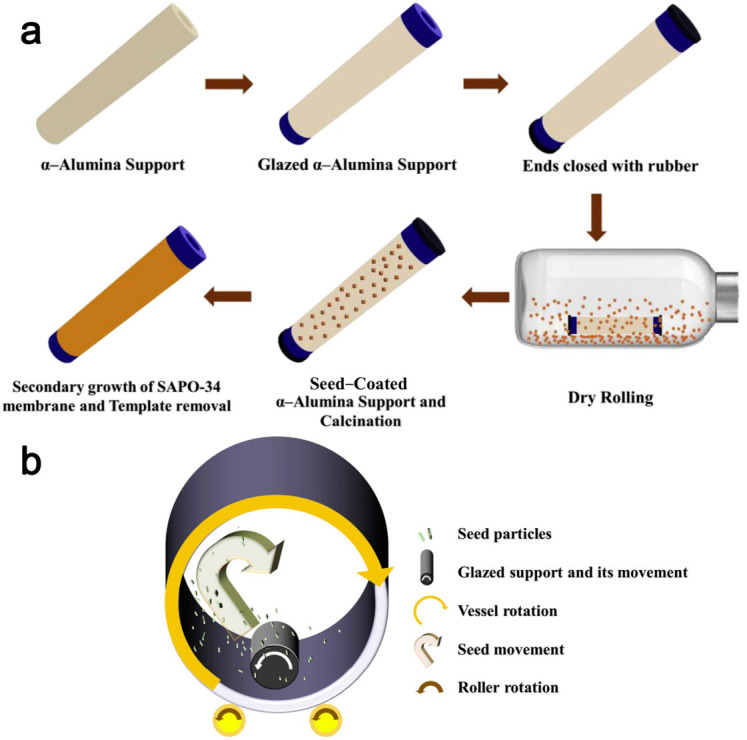
Schematic illustration of the membrane synthesis procedure (**a**); relative movement of all components during dry rolling (**b**). Reproduced with permission from reference [26]. Copyright 2020, Elsevier.

There are many advantages in the secondary growth process, including the ease of operation, controlled crystal orientation and shape, and the ability to adjust the film thickness, so as to achieve better reproducibility. All these benefits are directly linked to the pre-formed seed layer, making the seeding process a crucial element in controlling the microstructures of seeds and ensuring the reproducibility of the membrane during the secondary growth process. The secondary growth synthesis of SAPO-34 membranes has been widely reported in the literature [55,56,57]. However, the reliability of manual friction results have also been reported in the literature. Also, the widely used self-spin coating method has some limitations for commercial use, as the synthesis of the pipe film is difficult and not widely used in industrial processes. In 2020, Cho and colleagues introduced a new seeding technique called the dry rolling method for the secondary growth process used in the fabrication of SAPO-34 zeolite membranes [26]. As illustrated in Figure 6, this method involves passing seeds through a mechanical rolling support within a cylindrical roller containing dry seed particles, where SAPO-34 seeds are ground into smaller particles that are evenly distributed on the bracket and within its pores. Using a low-speed, long cylindrical roller results in a more uniform seed distribution. The seed layer is consistently spread due to the finely ground seeds, without causing any mechanical damage to the support. Seed coatings effectively develop SAPO-34 separation layers and demonstrate superior performance in subsequent processes compared to the conventional rubbing method used during growth. It is important to note that the dry rolling technique employs ball-milling to apply dried seeds onto the outer surface of the bracket. Consequently, this mechanical approach is expected to reduce human error in seed spreading and enhance the reliability of SAPO-34 zeolite membrane fabrication.

### 3.6. Molecular Layer Deposition

**Figure 7:**
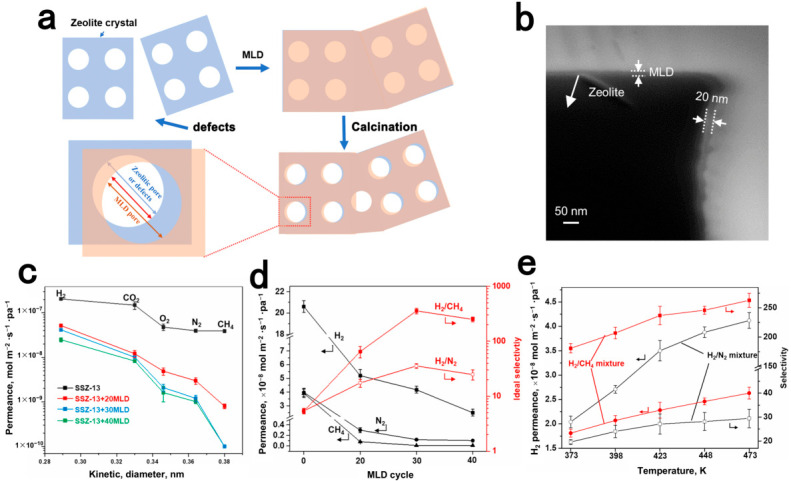
Schematic illustration of zeolite membrane synthesis through the molecular layer deposition method (**a**); TEM image of the zeolite membrane (**b**); separation performance of the SSZ-13 membrane for various gas separation (**c**), various molecular layer deposition cycles (**d**), and H_2_ permeance performance under various temperatures (**e**). Reproduced with permission from reference [33]. Copyright 2021, Elsevier.

The defects of zeolite membranes are almost inevitable, which greatly influence the reproducibility and performance. In addition, it is critical to fine-tune zeolite’s defects and pore structures to achieve accurate molecular separation. A molecular layer deposition strategy was explored to eliminate the surface defects of the zeolite membrane. The molecular layer deposition strategy uses the self-limited surface reaction to generate an organic–inorganic hybrid-type thin coating on various substrates, which has been proven to be an effective method for accurately controlling the membrane structure [58,59]. In addition, dense hybrids can be converted into porous layers through simple thermal treatment to remove organic compounds, thereby separating gas molecules. In 2021, Yu et al. further developed the molecular layer deposition (MLD) technology to synthesize SSZ-13 zeolite membranes by depositing an ultra-thin micro-pore coating (Figure 7) [33]. The MLD-modified SSZ-13 composite membrane could reduce the flow of non-selective defects/crystal pores, as shown in Figure 1. By comparing the separation performance of both the original membrane and the modified membrane to H_2_/N_2_ and H_2_/CH_4_ dual mixtures, the optimized MLD cycle significantly improved the selectivity of H_2_/N_2_ and H_2_/CH_4_ of the membrane. The optimized MLD cycle resulted in an ideal selectivity of H_2_/N_2_ and H_2_/CH_4_, reaching as high as 35.6 and 427, respectively, while the ideal selectivity of the base SSZ-13 film is about five. These observations showed that molecular layer deposition method is a promising technology that can accurately change the aperture of zeolite membranes and minimize the flow of non-selective defects for gas separation.

### 3.7. Orientation Crystal Seed-Induced Growth Method

**Figure 8:**
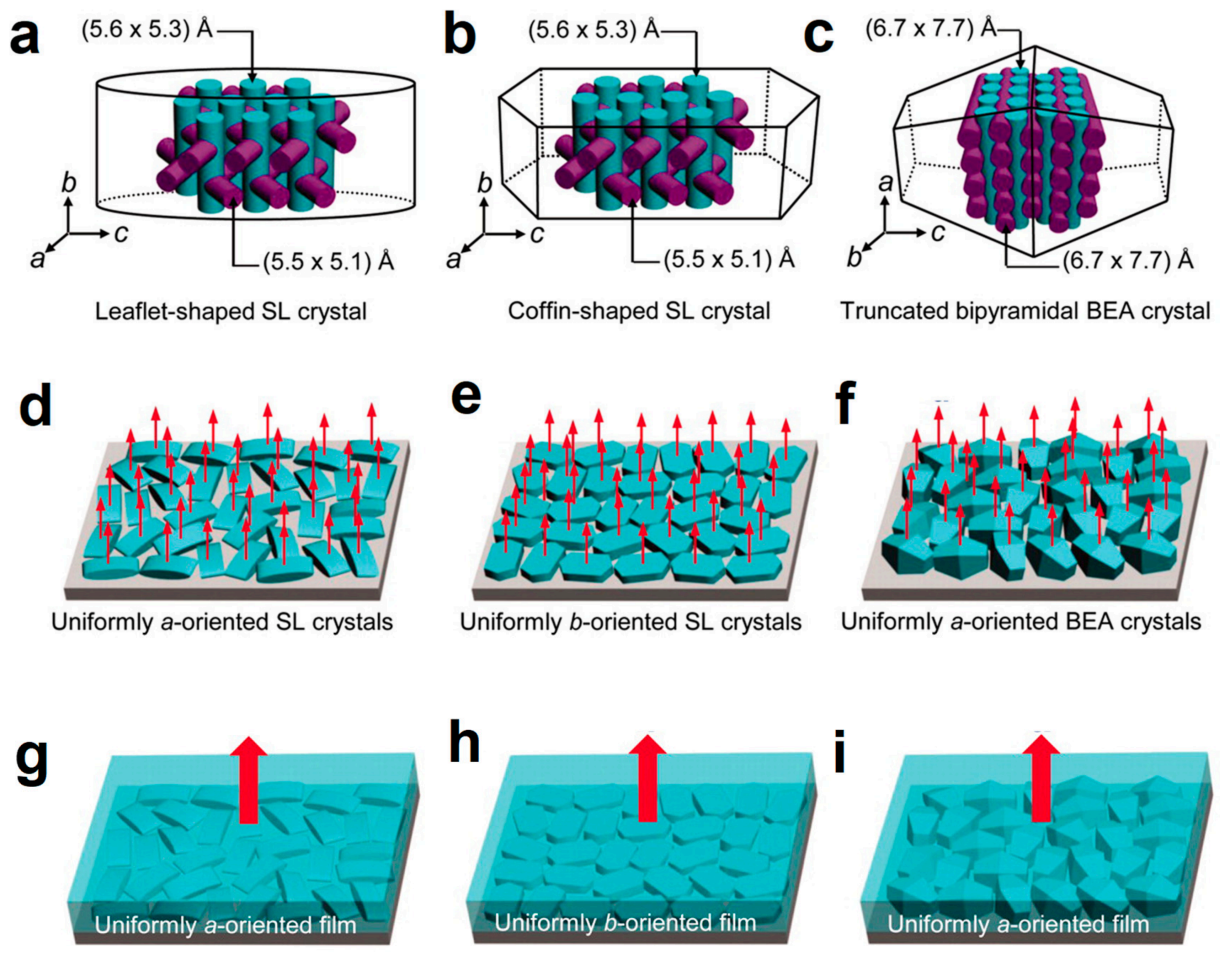
Schematic illustrations of various zeolites (**a**–**c**); representative a-oriented (**d**), b-oriented (**e**), and a-oriented monolayers (**f**); corresponding secondary growth of uniformly oriented films (**g**–**i**). Reproduced with permission from reference [23]. Copyright 2024, AAAS.

The performance of zeolite membranes can be greatly improved through the arrangement of the integrated channels, while the methods for membrane growth often introduce random pore channel directions along the substrate. Yoon et al. reported a simplified approach that can grow silicon-1 films and pure silicon-β-zeolite membranes on a substrate featuring uniform channels with a thickness of up to 8 μm. The precise gel composition and processing temperature are critical for promoting the secondary growth of the directional crystal layer on the substrate while inhibiting self-crystallization in the reaction medium. In their work, silicon-1 (SL) is a pure silica MFI zeolite, and its structure is shown in Figure 8a,b. Another type of Zee Beta (BEA) exhibits a truncated dual-cone shape, with 6.6 × 6.7 Å channels along the a (or b)-axis in a straight line and 5.6 × 5.6 Å channels running along the c-axis (Figure 8c) [23]. When trying to grow pure silicon BEA (SI-BEA) films on the substrate, no reports indicated that the a (or b)-axis is uniformly perpendicular to the thin film. In the case of the SL film, the leaflet-shaped (Figure 8a) and coffin-shaped (Figure 8b) crystals that were initially prepared on the appropriate substrate were oriented into an a-axial orientation. The authors have reported an improved membrane preparation method, which can repetitively a-oriented (Figure 8d,g) and b-oriented (Figure 8e,h) in SL and Si-BEA (Figure 8e,i) membranes on substrates.

### 3.8. Strategies to Reduce the Surface Defects

The removal of the template in the zeolite membrane usually causes defects, where the traditional high-temperature calcination process leads to shrinkage of the zeolite membrane and generates defects with poor selectivity [48]. Zhang et al. developed a mild template removal strategy by using an ozone-rich environment to effectively reduce thermal stress when removing the template, thereby producing high-quality ZSM-5 and DDR membranes [57]. Conversely, in wet oxygen conditions, the SAPO-34 membrane did not fully eliminate the thermal stress that occurs in the SAPO-34 film nor did the film completely remove any residual heat from conventional high-temperature treatment. The addition of water in an ozone atmosphere accelerated the oxidation of template molecules. The prepared SAPO-34 membrane had high permeability and high selectivity in CO_2_-CH_4_ separation, which was mainly due to its mild roasting conditions and high template-related efficiency related to wet oxygen. Water vapor as well as ozone causes the generation of hydroxyl radicals, which can penetrate into the pores of zeolite to effectively oxidize template molecules.

**Figure 9:**
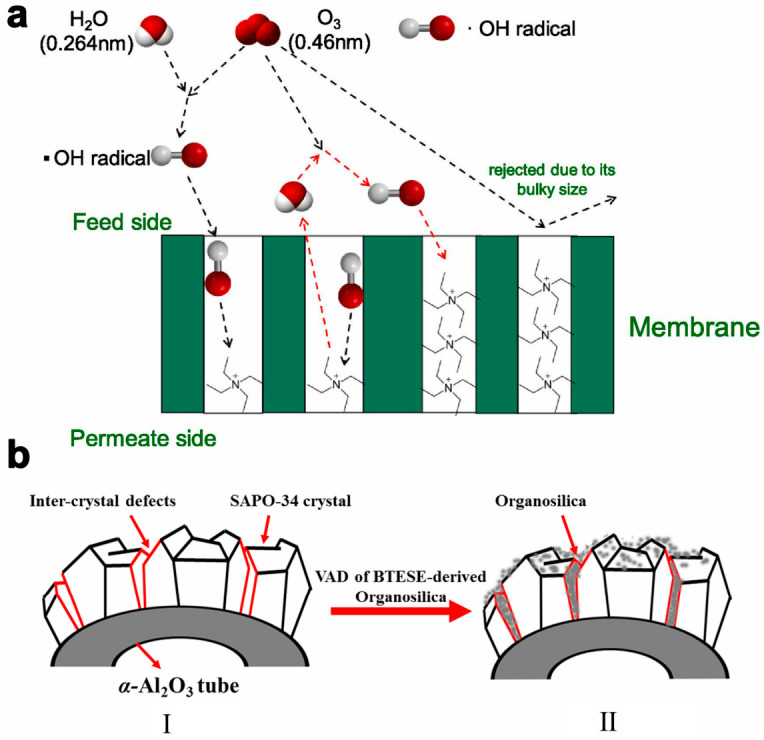
Schematic illustration of template removal under an ozone environment (**a**); schematic illustration of the organosilica deposition method to repair defects in SAPO-34 zeolite membranes (**b**). Reproduced with permission from references [56,57]. Copyright 2019, Elsevier.

For the removal of the templates from large- and middle-sized pore channels, the ozone molecules can diffuse into zeolite channels, where they can directly attack the template molecule given that their aperture is smaller than that of the medium and large pores. Heng et al. observed that when ozone is exposed to temperatures above 373 K, due to its instability, it may decompose into free radicals (Figure 9a). These free radicals can cause the following removal of templates in MFI zeolite films [60]. However, for template removal from small pores in zeolite (such as all-silicon LTA: 0.40 nm), the narrow pore entrance inside zeolite crystals hinders the direct reaction of molecular ozone and template molecules. It is hypothesized that small species with high oxidation capabilities, such as atomic oxygen and hydroxyl radicals, may be the main oxidant, because these species can diffuse to small zeolite channels.

Moreover, to repair this type of boundary defect, many post-treatment methods have been explored, such as chemical vapor deposition (CVD), molecular layer deposition (MLD), chemical liquid deposition (CLD), and so on [24,61,62]. Furthermore, in 2019, Hardacre proposed a simple vacuum-assisted deposition (VAD) method to address this challenge [56]. As shown in Figure 9b, the organic silica anchors on the SAPO-34 membrane, which supports the pores on the porosity aluminum through VAD, showing the performance of selectively separating CO_2_ from a CO_2_/CH_4_ mixture. In this method, organic silica was used to repair the non-selective defects in the SAPO-34 film without affecting the penetration of CO_2_ while significantly improving the selectivity of CO_2_/CH_4_. However, an excessive organic silicon coating on the SAPO-34 membrane via VAD can result in a thick layer, causing a significant decrease in CO_2_ penetration. In addition, a modified membrane tested with varying CO_2_ concentrations (20–50 mol%) in a CO_2_/CH_4_ gas feed indicates that the higher concentration of CO_2_ in the system can improve the separation performance of the membrane.

## 4. Advantages of Zeolite Membranes in Gas Separation

### 4.1. SAPO-34 Zeolite Membrane for CO_2_ Separation

**Figure 10:**
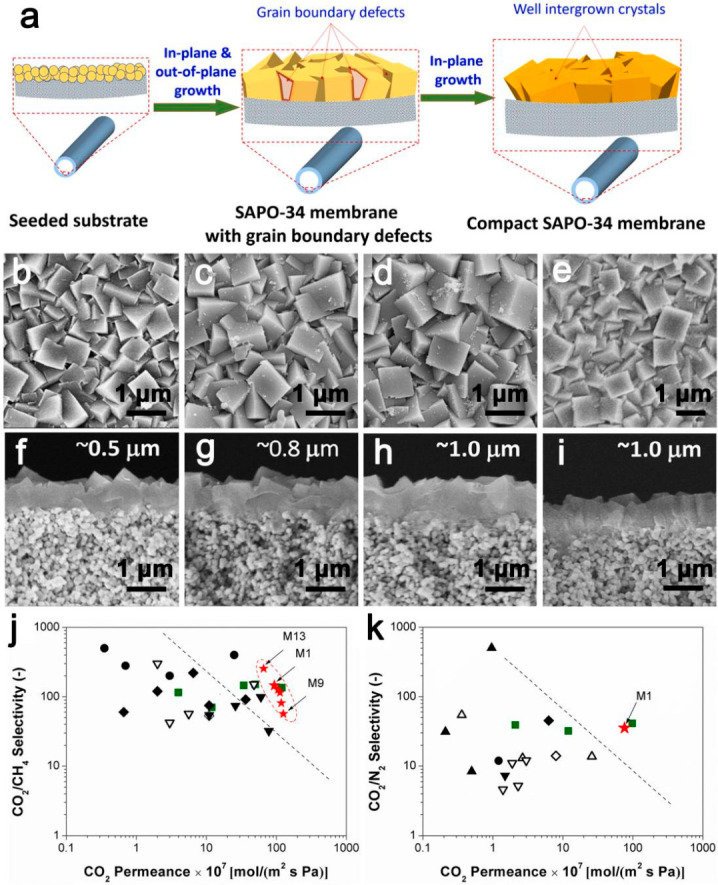
Schematic illustration for the preparation of thin SAPO-34 membranes (**a**); surface and cross-sectional SEM images of SAPO-34 membranes synthesized with different synthesis times: 12 h (**b**,**f**), 15 h (**c**,**g**), 18 h (**d**,**h**), and 24 h (**e**,**i**); comparison of separation performance of the current SAPO-34 membrane with other zeolite membranes for CO_2_/CH_4_ (**j**) and CO_2_/N_2_ (**k**) mixtures. 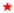: Thin SAPO-34 membranes, 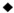: AlPO-18, 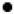: DDR, 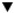: CHR, 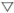: SSZ-13, 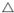: MFI, 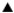: FAU, 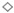: SAPO-17, 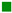: others SAPO-34. Reproduced with permission from reference [27]. Copyright 2022, Elsevier.

In order to achieve the effective separation of CO_2_/CH_4_ and CO_2_/N_2_, zeolite membranes, particularly the eight-membered zeolite membrane with a diameter of 3.6–4.0 Å, are ideal candidates. DDR-type zeolite films (3.6 Å × 4.4 Å) exhibit higher selectivity for CO_2_ over N_2_ and CH_4_ [29,63,64,65]. Wang et al. reported that the separation coefficient of CO_2_/CH_4_ of DDR zeolite membrane films was 500, and the CO_2_ permeance was 3.5 × 10^−1^ mol (m^2^·s·Pa)^−1^ [66]. Yu et al. [67] fabricated a thin DDR zeolite membrane with a thickness of 700 nm. This membrane delivered the optimal CO_2_/CH_4_ selectivity of 2325 and a high CO_2_ permeance of 34 × 10^−7^ mol/(m^2^·s·Pa) at −30 °C under a feed pressure of 3 bar. However, traditional DD3R zeolite membranes still face inherent fabrication drawbacks. Impurity crystals are readily formed during hydrothermal synthesis, and the traditional preparation route relies on toxic ethylenediamine solvents, severely restricting the practical application and industrial promotion of DD3R membranes. To address these limitations, Xue et al. [34] proposed a facile and eco-friendly synthesis protocol in 2025 for the fabrication of high-performance high-silica Sigma-1 zeolite membranes. Using sodium hydroxide as the mineralizer, the research systematically investigated the effects of template dosage and H_2_O/SiO_2_ molar ratio in precursor solutions on the morphological integrity and structural quality of as-prepared membranes. The optimal precursor molar composition was determined to be 9 ADA:60 SiO_2_:1 NaAlO_2_:3 NaOH:6000 H_2_O, which yielded a dense and defect-free Sigma-1 zeolite membrane with a thickness of 3.6 μm. Gas separation tests verified the prominent molecular sieving capability of the optimized membrane. The optimized membrane exhibited a CO_2_/CH_4_ separation selectivity of 304 and a CO_2_ permeance of 3.51 × 10^−8^ mol (m^2^·s·Pa)^−1^. Furthermore, the fabricated Sigma-1 membrane retained stable separation performance under both dry and humid operating conditions, proving its excellent environmental adaptability and long-term operational durability. Moreover, the eight-membered ring CHA zeolite, with a pore diameter of 3.83 × 3.8 Å, has become one of the most widely explored membrane materials as well [68]. Its pores are suitable for separating CO_2_, N_2_, and CH_4_. SSZ-13 and Si-CHA are aluminum silicate CHA-type zeolites, and SAPO-34 is a silicon aluminum phosphate CHA-type material. Moreover, in the process of hydrothermal synthesis, the thickness and quality of membranes are highly related to changes in synthetic time and temperature. The ideal state of membrane growth is to stop the expanding growth on the surface after the formation of a continuous layer. Therefore, a uniform, high-quality, and thin zeolite membrane can be produced with a large synthetic time. At the same time, it is necessary to improve the reproducibility and consistency of the membrane from each batch. In 2022, Wang et al. reported the fabrication of high-quality SAPO-34 zeolite membranes within a broad synthetic time window. The zeolite membrane could be controlled on brackets through secondary growth [27]. As shown in Figure 10, the nano-sized spherical SAPO-34 particles are uniformly deposited. When the SiO_2_/Al_2_O_3_ ratio is 300, the thickness and mass of the SAPO-34 membrane vary within the synthetic time window of 3 to 18 h. During large-scale production, larger synthetic time windows are related with the quality and uniformity of zeolite membranes. The typical membrane has a high CO_2_ penetration rate, and the separation selectivity of CO_2_/CH_4_ and CO_2_/N_2_ mixtures are 146 and 35, respectively. Furthermore, by using a more diluted synthetic solution, the H_2_O/Al_2_O_3_ ratio is 450. At a lower synthetic temperature of 453 K, the membrane thickness is reduced while maintaining a high quality when preparing SAPO-34 membranes. As shown in Figure 10, the SEM images show that the formation of the SAPO-34 membranes were synthesized using different methods, and a continuous SAPO-34 membrane with a thickness of about 500 nm was obtained after 12 h of hydrothermal synthesis. The separation performance of these membranes has been evaluated. As shown in Figure 10, the separation performance of typical membranes in the CO_2_/N_2_ mixture far exceeds that of other membranes [69], and the CO_2_ permeance of the existing membrane is 200–350 times that of the commercial membrane [70]. This work has shown that the current CHA film reached a high standard, and the super permeable and CO_2_-selective SAPO-34 membrane was an advanced membrane material with great potential for CO_2_ capture from natural and flue gas sources.

### 4.2. STT Zeolite Membrane for H_2_ Separation

**Figure 11:**
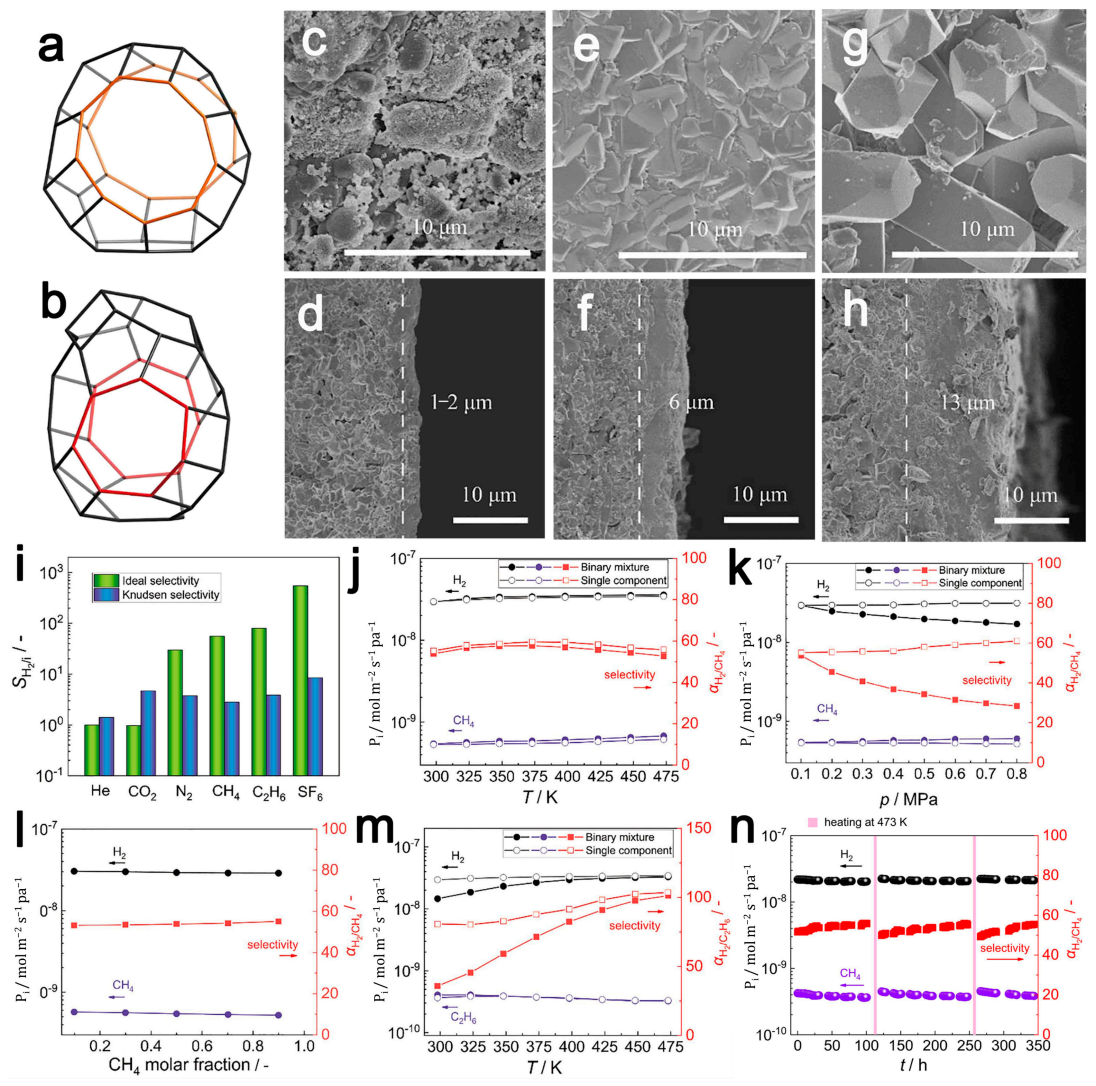
STT cage structures (**a**,**b**); SEM images of STT zeolite membranes at (**c**,**d**) 443 K (**e**,**f**) and 463 K (**g**,**h**); comparison of H_2_ selectivity (**i**); separation performance of H_2_/CH_4_ and H_2_/C_2_H_6_ under various parameters: temperature (**j**), pressure (**k**), CH_4_ molar fraction (**l**), and temperature-dependent performance (**m**); long-term stability test (**n**). Reproduced with permission from reference [35]. Copyright 2022, Elsevier.

STT zeolite possesses a cage-shaped structure connected to a two-dimensional window system, where it remains connected via seven-membered rings (7MRs) and nine-membered rings (9MRs). The effective size of the 7MR channel is 0.24 × 0.35 nm (Figure 11a), and the window size is 0.37 × 0.53 nm (Figure 11b). The pore-limiting diameter of a single STT unit is 0.37 nm, which is equivalent to the narrower side of the 9MR channel that is used as a fast transmission channel. As a result, the STT zeolite film is expected to increase the penetration rate of H_2_ and mitigate the disadvantages of competitive adsorption. Moreover, the H_2_ permeation rate through the STT zeolite film indicates its suitability for H_2_ separation at high temperatures [71]. Based on this, a silicon STT zigzag membrane was prepared for H_2_/CH_4_ separation from a fluorine-free environment (Figure 11) [35]. The crystallization cycle was shortened by 75% after the temperature was raised. After 2 days of hydrothermal synthesis, a H_2_/CH_4_ mixture was employed to evaluate the separation performance. The H_2_ permeance through the membrane reached 2.8 × 10^−8^ mol (m^2^·s·Pa)^−1^ and H_2_/CH_4_ selectivity reached 49.6 at 0.2 MPa and 298 K. Moreover, the STT zeolite membrane exhibited a long-term stability in the H_2_ separation cycles. More importantly, the STT membrane could resist CO and H_2_S to prevent them from entering the membrane. Specifically, this membrane remained stable for 336 h in a simulated furnace gas mixture with 100 ppm H_2_S. This will pave the way for the use of isolated hydrogen in practical applications.

### 4.3. Steam-Assisted Conversion of SAPO-34 Zeolite Membrane for H_2_ Separation

**Figure 12:**
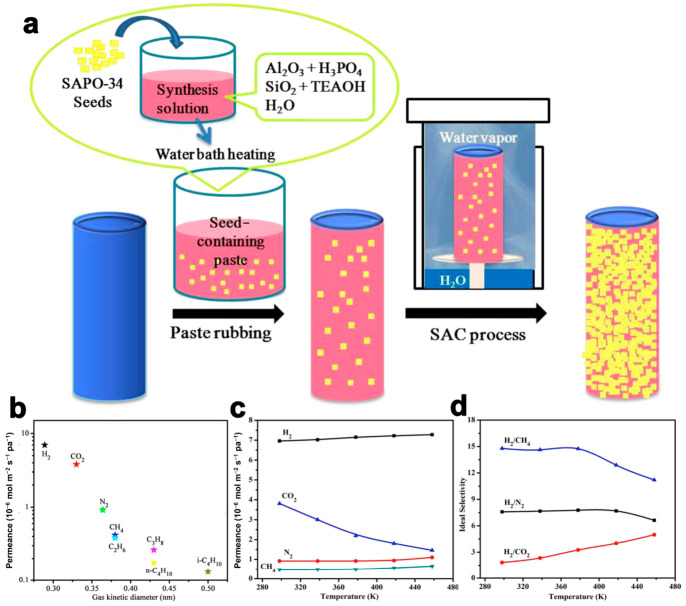
Schematic illustration of seed layer formation (**a**). Single-gas permeances of H_2_, N_2_, CO_2_, CH_4_, C_2_H_6_, C_3_H_8_, n-C_4_H_10_, and i-C_4_H_10_ for the SAPO-34 membrane, as a function of temperature with a pressure drop of 0.1 MPa, with separation factors (**b**), permeances (**c**), and ideal selectivity (**d**). Reproduced with permission from reference [72]. Copyright 2014, Elsevier.

To prepare H_2_ separation membranes, immersion, rubbing, brushing, and vacuum seeding methods have been widely used seeding strategies to grow zeolite on certain substrates. However, these methods have difficulty achieving a perfect seed layer on large-hole brackets. Yang et al. prepared SAPO-34 membranes by repetitive sample seeding using a steam-assisted conversion (SAC) technique [72]. As shown in Figure 12, SAC seeding involves depositing a type of solution on the substrate, and the SAC process transforms the solution into a dense and continuous seed layer. This strategy was able to obtain high-quality SAPO-34 membranes fabricated on low-cost, large-porous substrates, with a membrane thickness of ~4 μm. By using single gases such as H_2_, CO_2_, CH_4_, C_3_H_8_ and i-C_4_H_10_, the gas penetration of SAPO-34 membranes was examined. The results showed that the synthetic membrane exhibited high H_2_ permeability at room temperature (Figure 12b–d).

### 4.4. SAPO-34 Zeolite Membrane for SF_6_ Separation

**Figure 13:**
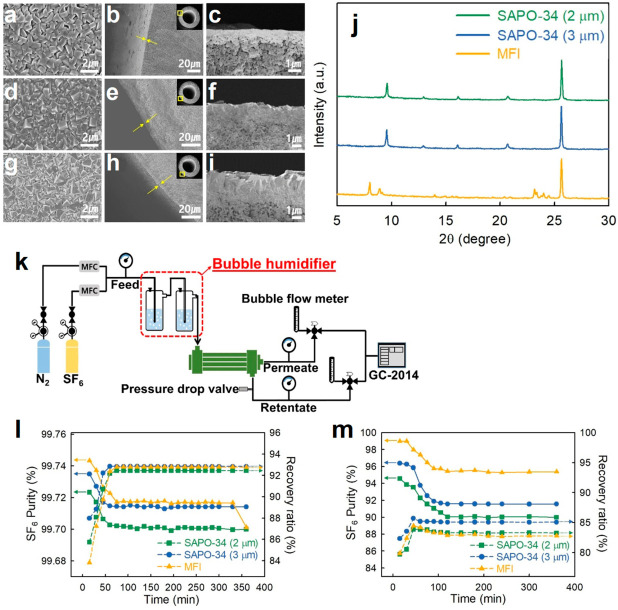
SEM images of various zeolite membranes of top views (**a**,**d**,**g**) and cross-sections (**b**,**c**,**e**,**f**,**h**,**i**); XRD patterns of the membranes (**j**); schematic illustration of the apparatus (**k**); SF_6_ purity and SF_6_ recovery rate (**l**); effect of moisture (**m**). Reproduced with permission from reference [73]. Copyright 2022, Elsevier.

The MFI zeolite membrane and SAPO-34 skeleton zeolite membrane have high gas permeability and high selectivity, which can be used to form a compact separation system. In 2022, Lee et al. fabricated MFI and SAPO-34 membranes to produce high-purity SF_6_ gas from SF_6_ exhaust gas (Figure 13) [73]. The membrane was synthesized on hollow alumina fiber carriers, and the gas diffusion performance of mixed-gas and single-gas components in N_2_/SF_6_ mixtures was evaluated. Subsequently, by using a mixture of 10% N_2_/90% SF_6_ and 20% N_2_/80% SF_6_, an SF_6_-enrichment experiment was performed to obtain 99.7% SF_6_ gas. As shown by this work, ultra-pure SF_6_ gas (>99.7%) produced by a single zeolite membrane that met the reusable guidelines of the corresponding device. Generally, it is difficult to achieve this purity level with only a polymerization film. The synthetic MFI film has high N_2_ permeability (about 2000 gas permeability units) and low N_2_/SF_6_ selectivity (<50) under 2 bar. Meanwhile, the SAPO-34 membrane shows the opposite trend—that is, high N_2_/SF_6_ selectivity (>500) and relatively low transparency (<600 gas permeability units). It is interesting to note that the SF_6_ gas can be effectively enriched by the MFI membrane system, while the SAPO-34 membrane needs to reduce the feed pressure to achieve the same purity by reducing the inlet flow. Moreover, it was found that the MFI membrane, with an area of 0.088 m^2^, is enough to purify all the SF_6_ gas generated by the related device under 145 kV within 6 h, indicating that there is an opportunity to achieve a compact SF_6_-enrichment system.

Through the control of feed pressure, permeate classification, and feed flow, the MFI and SAPO-34 membranes enhanced the enrichment effect on SF_6_. The ideal hybrid model was analyzed to explain the enrichment dynamics of the membrane for SF_6_ enrichment. Therefore, considering its small size with a high separation performance of the separation system, the MFI membrane represents a very promising choice for industrial purification of waste SF_6_ gas.

### 4.5. SSZ-13 Zeolite Membrane for CO_2_ Separation

SSZ-13 is a type of aluminum silicate with a three-dimensional structure and high affinity towards CO_2_, even in the presence of water, making it promising for CO_2_/CH_4_ and CO_2_/N_2_ separation membranes [74,75,76]. Research has shown that SSZ-13 membranes have good performance and high thermal and chemical stability. Kosinov et al. prepared a high-silicon SSZ-13 membrane with a diameter of 300 nm on the inner surface of an aluminum hybrid fiber support [77,78]. The permeance of CO_2_ was about 3 × 10^−7^ mol (m^2^·s·Pa)^−1^ under a pressure of 600 kPa. They further reported good performance of the low-silicon membranes during the separation of an ethanol/water mixture. In addition, high-silicon membranes showed more effective performance in the separation of gas mixtures, such as CO_2_/CH_4_. Moreover, it was demonstrated that the gas permeation of the amphiprotic membrane decreases in the proportion with the increase in SiO_2_/Al_2_O_3_ in the synthetic gel. Pelin et al. also prepared non-defective, high-silicon SSZ-13 membranes on porous aluminum oxide carriers by optimizing the parameters of seed concentrations [28], coating methods, synthesis times, and templating processes. The CO_2_/CH_4_ selectivity of the membrane was 25–30, and the transmission rate of CO_2_ was 2 × 10^−7^ mol (m^2^·s·Pa)^−1^ under a pressure of 200 kPa and a temperature of 22 °C. In comparison, the study conducted by Peng et al. [79] in 2024 achieved substantial breakthroughs in synthesis reproducibility, high-pressure operation stability, and scalable manufacturing of CHA-type zeolite membranes. Different from conventional sol–gel seed synthesis, the team proposed an inter-zeolite conversion strategy by using commercial FAU zeolite as the sole Si/Al source to prepare SSZ-13 seeds, which yields a shortened induction period and an extended crystallization phase. This seed design grants the growing membrane a prominent self-healing ability for non-selective inter-crystalline pores, enabling a consistent membrane thickness of ~3 μm across an ultra-wide synthesis time range of 24–96 h. More notably, extending the hydrothermal treatment can steadily elevate the separation selectivity without causing permeance decay, which effectively broadens the originally narrow synthesis window and greatly improves batch-to-batch consistency. The optimized hollow fiber SSZ-13 membrane retained a CO_2_/CH_4_ selectivity of 126 with a high permeation flux at a feed pressure up to 6.1 MPa, well satisfying the demands of industrial natural gas upgrading. However, the effects of different dual mixtures have not been fully explored, which is exactly the challenge that needs to be addressed.

**Figure 14:**
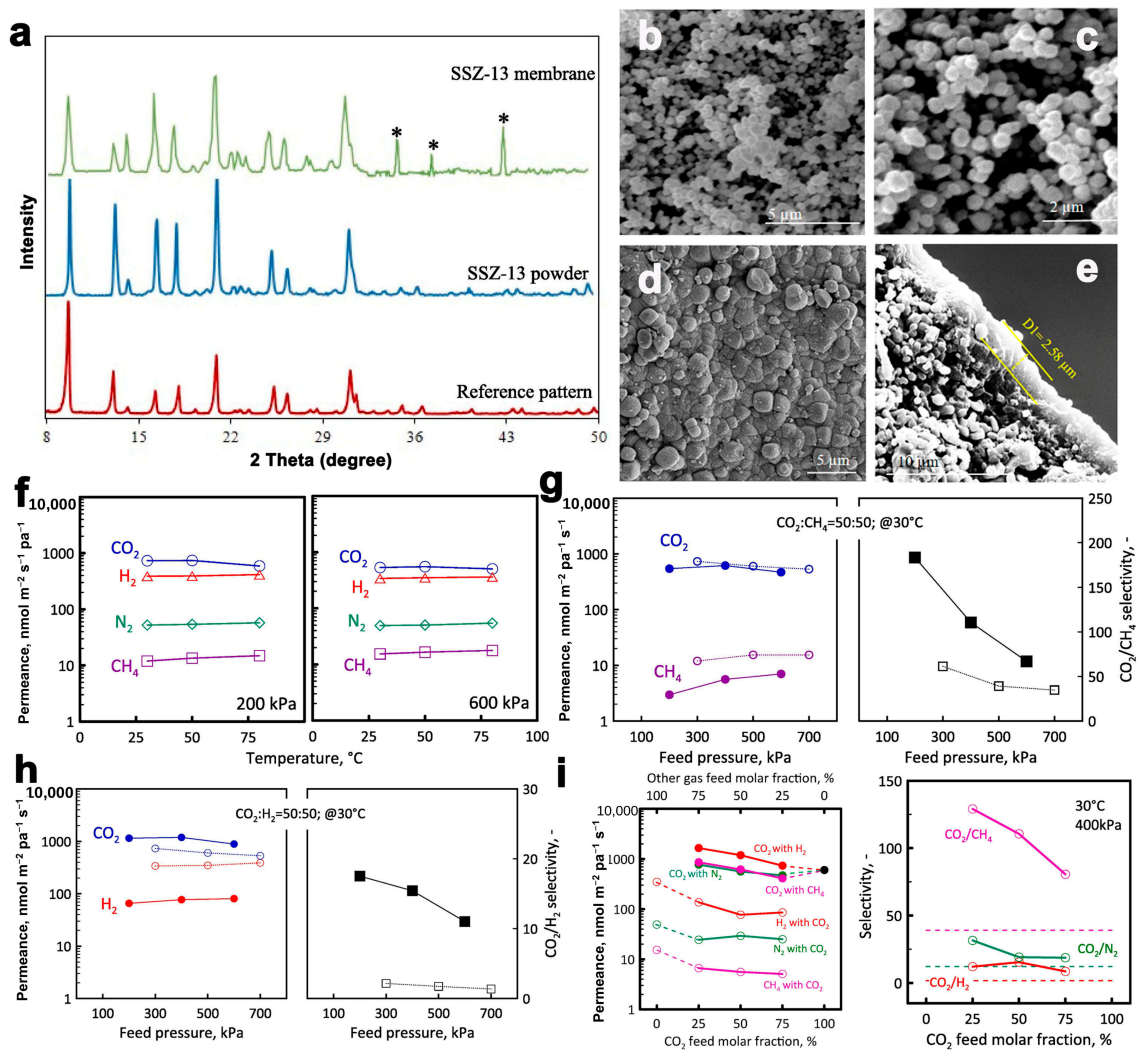
XRD patterns (**a**); SEM images (**b**,**c**), top view (**d**), and cross-section (**e**) of the SSZ-13 membrane; single-gas permeance (**f**); permeance and selectivity of CO_2_ and CH_4_ (**g**); permeance and selectivity of CO_2_ and H_2_ (**h**); influence of CO_2_ feed molar fraction (**i**). Reproduced with permission from reference [29]. symbol (*): α- Alumina support. Equimolecular mixture (full symbols) and single gas (empty symbols). Copyright 2021, Elsevier.

Maghsoudi et al. prepared SSZ-13 membranes on α-aluminum supports (Figure 14) through a newly developed synthesis method for separating CO_2_ from light gases, such as CH_4_, N_2_, and H_2_ [29]. The performance of the zeolite membranes was evaluated in a single-gas environment, with a pressure of 600 kPa and a temperature of 30–80 °C. Under different feed pressures (up to 600 kPa) and different feed compositions, the performance under mixed-gas conditions and the interaction of gas permeation were analyzed. It was found that the permeance of single-gas CO_2_ was improved, while the selectivity of CO_2_/CH_4_ mixture was increased by three times. Moreover, it was observed that the selectivity of CO_2_/N_2_ and CO_2_/H_2_ increased by more than five times. For other components, the mixed-gas selectivity of CO_2_/CH_4_, CO_2_/N_2_, and CO_2_/H_2_ decreased, but it always maintained a higher selectivity for gases with higher permeation rates than that of a single gas (Figure 14f–i).

### 4.6. Increasing the Separation Selectivity in CHA Zeolite Membranes by Adding a Third Gas

It has been reported that adding a third gas in a gas mixture can greatly promote separation selectivity [80]. For example, Nicolas et al. reported that when propane is added to the raw material, the permeance of the MFI membrane (0.55 nm) is reduced by 75%, and the CO_2_/N_2_ separation selectivity increased from three to six [81]. Although they did not explain the increase in selectivity, it may be attributed to the expansion of MFI crystals. It has been shown that when a linear hydrocarbon compound is adsorbed in MFI zeolite, the expansion of the MFI crystal reduces the size of defects, thereby decreasing permeance through non-selective defects. If the majority of the target gases permeate through non-selective defects, then the reduction in permeance through defects leads to an increase in CO_2_/N_2_ selectivity. Moreover, Himeno et al. demonstrated that by adding 1% propyne to a CO_2_/CH_4_ mixture only slightly reduces the penetration of CO_2_ through the DDR membrane, which maintain the selectivity of CO_2_/CH_4_ at the same time [82]. It is difficult for propane to enter DDR pore channels, and any changes in permeation are probably due to the adsorption on the outer surface of the membrane support [83]. Moreover, Baertsch et al. studied the penetration of ternary aromatics mixtures through MFI membranes [84]. The penetration efficiency of all hydrocarbons slowed down to that of the slowest component. Obviously, the MFI channels were narrow enough that the aromatic compounds could not pass through easily.

It is interesting to note that adding a low concentration of propane to the H_2_/N_2_ mixture can improve the separation performance the SAPO-34 membrane by reducing the N_2_ permeation rate to more than that of H_2_. Because propane diffuses into SAPO-34 pores, it takes more than 2 days to reach a stable state, while their adsorption effect is strong, thereby reducing the permeance of H_2_ and N_2_. Although the permeance of the weaker H_2_ is reduced, the penetration rate of N_2_ is reduced significantly more than the permeation rate of H_2_. This is because the diffusion rate of larger N_2_ molecules decreases more. At 298 K, the addition of propane increases H_2_/N_2_ selectivity, while with the temperature increases, the effect on selectivity decreases because the adsorption of propane in SAPO-34 pores is reduced. Ethylene also reduces the permeation rates of H_2_ and N_2_, but it does not change the selectivity of H_2_/N_2_. The impact of smaller ethylene on N_2_ is smaller than that of propane, so the overall effect is that selectivity remains unchanged. After removing alkanes from the feed, the adsorption effect of the SAPO-34 membrane reverses.

## 5. Summary and Outlook

In summary, with the active exploration of novel microporous materials and preparation techniques, microporous membranes have achieved significant progress in separation performance, thermal stability, and chemical stability. Zeolite membranes have changed the gas separation processes greatly, which offers significant advantages compared with traditional methods. Through surface modification, the permeance of zeolite membranes has been significantly enhanced, surpassing levels a decade ago by an order of magnitude. With ongoing advancements in materials science, fabrication techniques, and process integration, these membranes are anticipated to play a critical role in gas separation, as well as in tackling energy and environmental challenges.

Therefore, in this work, we review the recent representative research work on the fabrication strategies of zeolite membranes and their applications in gas separation. As is shown in this work, much evidence has shown that zeolite materials have extraordinary performances, particularly in gas separation due to their uniform microporous structures, as separation performance mainly relies on the pore properties. In this review, we summarize the most critical fabrication techniques in preparing uniform zeolite membranes and the strategies to minimize surface defects. These fabrication techniques include the self-limiting growth method, multi-parameter-controlled fabrication, “gel nuclei-less” route, gel-modulated strategy, dry rolling method, molecular layer deposition, molecular layer deposition, etc.

Subsequently, we summarize the formation mechanisms and strategies regarding the control of pore alignment on the membrane surface and the methods that minimize defects on the membrane. In addition to the above achievements, attempts have been made to design zeolite membranes with controlled pore properties, as well as the corresponding potential for unique separation performance.

Moreover, the synthesis of new types of zeolite membranes is also challenging in terms of large-scale synthesis and low-cost production, as well as hetero-component doping and modification. Accurate control of the alignment of the pore channels of zeolite materials on the membrane surface poses a great challenge as well, as zeolite materials can provide several pore alignments and corresponding separation properties. In addition, the relationship between the porous structure of zeolite membranes and corresponding separation performance has not been explored thoroughly. For example, the interaction between different pore channels and gas molecules may greatly influence the separation performance. These mechanisms have not been extensively studied due to their complicated interactions between pore cages, pore channels, and small gas molecules. Moreover, strategies are highly needed to reduce the surface defects of zeolite membranes, which is as important as the zeolite type for separation performance.

Furthermore, we reviewed the representative gas separation applications, and the impact of the zeolite topological structure/chemical composition on the gas separation performance was analyzed. It is noted that the diffusion and transportation of target molecules are more complicated than those of hydrocarbon compounds. For zeolite membranes, surface diffusion is generally accepted as the dominant mechanism for gas transmission; however, no models can fully elucidate the complex gas transportation behavior, which needs to be further explored.

In view of the significant improvement in inorganic microporous zeolite membranes in terms of gas separation performance, hydrophilicity, and chemical elasticity, it is expected that the next step will involve evaluating their performance under industrial-scale conditions. To accelerate this translation from laboratory research to industrial application, targeting the current bottlenecks of poor batch reproducibility, high manufacturing costs, and insufficient long-term stability in complex industrial environments, future research should be focused on artificial-intelligence- and machine-learning-assisted rational membrane design and synthesis process optimization, low-cost scalable fabrication, long-term stability evaluation under practical operating conditions, and the development of zeolite-based hybrid membrane technologies, so as to further promote the large-scale application of zeolite membranes in gas separation.

## Figures and Tables

**Table 1:** Summary of different zeolite membrane types in terms of their synthesis method, gas separation application, permeability, selectivity, and advantages and limitations.

Zeolite Membranes	Synthesis Method	Gas Separation Application	Permeability mol·m^−2^·s^−1^·Pa^−1^	Selectivity	Advantages	Limitations	Ref.
SAPO-34 zeolite membrane	Multi-parameter-controlled fabrication	CO_2_	~5.0 × 10^−6^	CO_2_/CH_4_ (152)	Ultra-thin and highly permeable	Performance drop at high pressure	[25]
SSZ-13 zeolite membrane	Multi-parameter-controlled fabrication	CO_2_	~6.3 × 10^−7^	CO_2_/H_2_ (161)	Universal applicability	Complex variable coupling	[28]
LTA zeolite membrane	Self-limiting growth method	H_2_	1.5 × 10^−6^	H_2_/CO_2_ (20.9)	Thin and dense	Stringent process	[30]
				H_2_/CH_4_ (17.3)			
SAPO-34 zeolite membrane	“Gel nuclei-less” route	CO_2_	1.0 × 10^−5^	CO_2_/CH_4_ (135)	Defect-free with high permeance	Performance drop at high pressure	[31]
	Gel-modulated strategy	H_2_	7.6 × 10^−7^	H_2_/N_2_ (23.1)	Highly reproducible	Complex process	[32]
SSZ-13 zeolite membrane	Molecular layer deposition	H_2_	4.27 × 10^−8^	H_2_/N_2_ (35.6)	Pore tuning and defect control	Difficult to scale-up production	[33]
				H_2_/CH_4_ (427)			
Sigma-1 zeolite membrane	Secondary growth	CO_2_	3.51 × 10^−8^	CO_2_/CH_4_ (304)	Stable and eco-friendly	High cost and complex process	[34]
STT zeolite membrane	Secondary growth	H_2_	2 × 10^−8^	H_2_/CH_4_ (49.6)	Stable and eco-friendly	High cost and complex process	[35]

## Data Availability

No new data were created or analyzed in this study. Data sharing is not applicable to this article.
